# Gender, trauma type, and PTSD prevalence: a re-analysis of 18 nordic convenience samples

**DOI:** 10.1186/1744-859X-11-26

**Published:** 2012-10-29

**Authors:** Daniel N Ditlevsen, Ask Elklit

**Affiliations:** 1 National Centre for Psychotraumatology, University of Southern Denmark, Campusvej 55, Odense M, DK, 5230, Denmark

**Keywords:** Posttraumatic Stress Disorder (PTSD), Gender differences, Trauma types

## Abstract

**Background:**

The aim of the study was to examine a possible trauma type related variance in the gender difference of posttraumatic stress disorder (PTSD) prevalence.

**Methods:**

An analysis was conducted on 18 convenience sample studies including data from a total of 5220 participants. The studies all applied the Harvard Trauma Questionnaire – part IV to assess PTSD. Cohen’s *d* was used to measure variance in gender differences. Trauma types included disasters and accidents, violence, loss, chronic disease and non-malignant diseases.

**Results:**

The results showed an overall gender difference in PTSD prevalence similar to previous findings. Thus, women had a two-fold higher prevalence of PTSD than men. Besides categorical analyses, dimensional analyses of PTSD severity were also performed; the latter were associated with twice as large effect sizes. Females were more vulnerable to PTSD after disasters and accidents, followed by loss and non-malignant diseases. In violence and chronic disease, the gender differences were smallest.

**Conclusions:**

The findings support the existence of a trauma type related variance in gender differences in PTSD prevalence.

## Background

Epidemiological studies have repeatedly found that men and women differ in their risk of trauma exposure and in their risk of Posttraumatic Stress Disorder (PTSD). Men are at higher risk of being exposed to traumatic events during their lifetime compared to women; and women are at higher risk of developing PTSD than men. The National Comorbidity Survey (NCS) by Kessler and co-workers [1] studied the exposure of trauma in a nationwide probability sample of 5877 adult residents of the United States and found that 61% of men and 51% of women had experienced at least one traumatic event during their lives.

Kessler and colleagues [1] found that the estimated lifetime prevalence of PTSD in the NCS was 7.8%. Females showed a prevalence of 10.4% and men a prevalence of 5.0%. The gender difference in prevalence of PTSD remained when controlling for type of event. However, women in the NCS were more likely than men to experience a trauma which has a high probability of precipitating PTSD, for example, *sexual assault or sexual molestation.* The event with the highest conditional risk of developing PTSD was *rape*, which was reported far more often by females than males. However, relatively few studies have compared the prevalence of PTSD and gender differences across different trauma types.

### Trauma types and gender differences

A trauma type is an overarching category for a series of different individual trauma events that are related or familiar to each other through some defining similar characteristics. For example, earthquakes, floods, volcanic eruptions, avalanches, and hurricanes could all be included under the same trauma type (*natural disaster*) because they are all natural occurrences which are outside the control of man and which often lead to the massive destruction of property and numerous casualties. It is important to note that trauma types can be categorized both broadly and narrowly, which impacts variance within categories and comparability between different studies. Previous studies have assessed gender differences in PTSD in association to different trauma types. The studies showed different results for trauma type related gender differences in PTSD prevalence. Tolin and Foa [2] studied gender differences in the prevalence of specific types of traumatic experiences. They divided traumas into nine different types and made comparisons of gender differences in the frequency of these different trauma types. A gender difference was present for some trauma types while others showed no gender difference in the prevalence of specific types of potentially traumatic events (PTE). Comparisons for PTSD were also made. Across all studies, the results concluded that the frequency and severity of PTSD was greater among women than among men (*d* = 0.29) [2]. The trauma type which showed the highest gender difference in PTSD prevalence based on the combined effect size for diagnosis and severity was *nonsexual assault*, where females had a significantly and robustly higher risk of PTSD diagnosis than men (*d* = 0.65). The trauma types of *combat, war, or terrorism, nonsexual child abuse or neglect,* and *adult sexual assault* did not indicate a significant difference between male and female participants in the prevalence of PTSD. Stein, Walker, and Forde [3] reported that the prevalence of PTSD was higher for women than for men. They used three broad types of trauma when they examined the conditional probabilities of full or partial PTSD in men versus women following exposure to trauma. The trauma types were *sexual traumatisation, nonsexual assaultive violence,* and *non-assaultive trauma.* The study showed that women were at an increased risk for current PTSD after *nonsexual violent assaultive trauma* but not after *non-assaultive trauma.*

Knowledge of the trauma type related variance of gender differences in PTSD prevalence could contribute to the aetiology and phenomenology of PTSD. The purpose of the present study is to examine the trauma type related variance of gender difference in PTSD. Knowledge of how gender differences in PTSD prevalence potentially vary in relation to different trauma types could contribute to the aetiology and phenomenology of PTSD. Thus, we find it relevant to examine the following hypotheses concerning gender and trauma types in PTSD: (a) an overall gender difference in PTSD prevalence will be found in the included convenience samples, showing that women are more vulnerable to PTSD than men; (b) all included trauma types will show a gender difference in PTSD prevalence with women showing a higher prevalence than men; (c) the gender difference in PTSD prevalence will vary from trauma type to trauma type. Thus, the aim of the present study is to examine whether women compared to men show a higher level of PTSD in relation to all of the included trauma types.

## Methods

### Procedure and participants

A large sample of PTSD studies conducted in Denmark or in Iceland in cooperation with the senior author was reviewed to find eligible studies for the present study. The preliminary data consisted of 46 different studies with data from a total of 13507 participants. Studies were selected for inclusion by the following criteria: (a) the study was a convenience study examining traumatization or PTSD in relation to a specific trauma event (e.g. being exposed to an earthquake, or a robbery); (b) the study included both male and female participants; (c) the study used the Danish or the Icelandic version of the Harvard Trauma Questionnaire (HTQ) [4]. For the studies included, criteria were also set up for participant inclusion. The criteria in this respect were (a) the participants have notified their gender; (b) they are at least 13 years of age; (c) the participants have given full information on the HTQ. The data consequently consisted of data from eighteen different convenience studies of trauma and PTSD conducted from 1996 to 2008 [5-22]. The sample was composed by 5220 participants, 2101 (40.2%) men and 3119 (59.8%) women. The age distribution of the participants ranged from 13 to 87 years of age; the mean age was 38.0 (*SD* = 13.55). Fifteen of the included studies were carried out in Denmark and three in Iceland. Thus, the majority of the participants (4600) were Danish. The remaining participants (620) were of Icelandic origin. Studies within the hospital sector were approved by a regional Helsinki committee. Data collection in all other sectors was undertaken in accordance with the Nordic ethical rules for psychologists.

The present study examined gender differences in the prevalence of PTSD in relation to five trauma types. The five trauma types were constructed based on the studies included for analysis. The categories of trauma types were: (a) being exposed to a disaster or an accident (*disaster and accident*); (b) experiencing the loss of a close relative or friend (*loss*); (c) experiencing a malignant or severe chronic disease to oneself or to a close relative (*chronic disease*); (d) experiencing a non-malignant disease to oneself or a close relative (*non-malignant disease*); and (e) being exposed to or witnessing violence (*violence*). The trauma types, *loss, chronic disease,* and *non-malignant disease* have previously been very sparsely represented under broader categories in studies examining trauma types e.g. [23].

### Measures

The Danish version of the HTQ was used in 15 of the studies and an Icelandic version was used in the remaining three studies. The Danish version of the HTQ has been found to be both valid and reliable [24]. The HTQ can be used for estimating a PTSD diagnosis through measuring the severity of PTSD symptoms. The HTQ originally consisted of 30 items. Some newer studies divided item 16 (sudden emotional or physical reactions when reminded of the incident) into two items. However, this additional item was not included in the HTQ total scores used for analysis. The sixteen items of the HTQ were distributed across the three DSM-IV subscales of PTSD in that avoidance comprised seven items, re-experiencing comprised four items, and arousal comprised five items. The items were scored on a four-point Likert scale (1 = not at all; 4 = extremely). Besides severity, a diagnosis can be estimated through an algorithm. Only scale items above or equal to 3 on the HTQ were considered for a PTSD diagnosis. For the full PTSD diagnosis one symptom of re-experiencing, three symptoms of avoidance, and two symptoms of arousal were needed. For participants falling short of the full PTSD diagnosis by missing one symptom a sub-clinical level diagnosis of PTSD was given. The original study by Mollica et al. found good reliability and validity for the scale and found that the HTQ self-report measure of PTSD had 88% concordance with interview based estimates of PTSD [4]. Furthermore, participants’ information regarding gender and age was considered.

### Statistical analyses

Descriptive analyses were performed on the data using mean scores, standard deviation (SD), and percentages. One-way analyses of variance (ANOVA’s) with descriptive statistics were performed to compare the dichotomous independent variable of gender and the continuous dependent psychometric variable (HTQ-total). Additionally, effect sizes (Cohen’s d) were calculated for gender differences in PTSD severity scores with d = .2 indicating a small, d = .5 indicating a medium, and d = .8 indicating a large effect size, respectively [25].

## Results

### Prevalence of PTSD

The results regarding prevalence of PTSD can be seen in Table 1. The number of participants who qualified for PTSD in the total sample was 1075 participants of 5220, which means 20.6%. The female participants showed a nearly two-fold higher prevalence of PTSD (25.6%) than the male participants (13.2%). As Table 1 shows, the percentage of participants who qualified for PTSD varied within the different trauma types. *Disaster and accident* was the trauma with the highest prevalence of PTSD with a prevalence of 25.7%. *Loss* and *violence* showed a prevalence of PTSD by 19.7% and 19.0%, respectively. *Non-malignant disease* showed a prevalence of 13.9%, whereas, *chronic disease* was the trauma type which showed the lowest prevalence of PTSD with a prevalence of 7.5%.

**Table 1:** PTSD Prevalence and HTQ total score

Study *	**Exposure N**			**Prevalence of PTSD N (%)**			**PTSD M (SD) effect size**				**HTQ total M (SD) effect size**			
	**Male**	**Female**	**Total**	**Male**	**Female**	**Total**	**Male**	**Female**	***d***	***r***	**Male**	**Female**	***d***	***r***
**Disaster and accidents**	960	1435	2395	139 (14.5)	476 (33.2)	615 (25.7)	0.1 (0.4)	0.3 (0.5)	−0.45	−0.22	49.1 (17.4)	63.5 (16.9)	−0.84	−0.39
Earthquake victims [5]	33	40	73	3 (9.1)	6 (15.0)	9 (12.3)	0.1 (0.3)	0.2 (0.4)	−0.18	−0.09	42.7 (14.1)	48.0 (13.1)	−0.39	−0.19
Explosion affected residents [6]	233	236	469	18 (7.7)	41 (17.4)	59 (12.6)	0.1 (0.3)	0.2 (0.4)	−0.29	−0.15	46.3 (11.5)	53.4 (13.5)	−0.57	−0.27
Rescue personnel at explosion [7]	398	28	426	7 (1.8)	0 (0.0)	7 (1.6)	0.0 (0.1)	0.0 (0.0)	0.19	0.09	38.4 (8.4)	40.6 (9.6)	−0.25	−0.12
Whiplash victims [8]	296	1131	1427	111 (37.5)	429 (37.9)	540 (37.8)	0.4 (0.5)	0.4 (0.5)	−0.01	−0.00	66.3 (17.3)	66.7 (16.2)	−0.02	−0.01
**Loss**	501	804	1305	65 (13.0)	192 (23.9)	257 (19.7)	0.1 (0.3)	0.2 (0.4)	−0.28	−0.14	27.6 (8.2)	31.7 (9.4)	−0.47	−0.23
Cancer patient relatives** [9]	61	175	236	23 (37.7)	78 (44.6)	101 (42.8)	0.4 (0.5)	0.4 (0.5)	−0.14	−0.07	35.1 (7.1)	37.9 (8.4)	−0.35	−0.17
Elderly bereaved [10]	20	38	58	3 (15.0)	16 (42.1)	19 (32.8)	0.2 (0.4)	0.4 (0.5)	−0.62	−0.30	43.4 (13.5)	66.4 (20.8)	−1.31	−0.55
Elderly bereaved (prospective study) [11]	113	183	296	15 (13.3)	31 (16.9)	46 (15.5)	0.1 (0.3)	0.2 (0.4)	−0.10	−0.05	49.5 (12.0)	52.0 (13.5)	−0.20	−0.10
Parents who have lost an infant *** [12]	43	55	98	3 (7.0)	15 (27.3)	18 (18.4)	0.1 (0.3)	0.3 (0.4)	−0.55	−0.27	50.3 (12.5)	62.7 (14.1)	−0.93	−0.42
Parents who have lost an infant **** [13]	264	353	617	21 (8.0)	52 (14.7)	73 (11.8)	0.1 (0.3)	0.1 (0.4)	−0.21	−0.11	47.0 (13.0)	53.5 (15.9)	−0.45	−0.22
**Chronic disease**	179	368	547	11 (6.1)	30 (8.2)	41 (7.5)	0.1 (0.2)	0.1 (0.3)	−0.08	−0.04	46.0 (12.8)	51.3 (14.0)	−0.39	−0.19
Families with chronically ill children [14]	32	53	85	5 (15.6)	7 (13.2)	12 (14.1)	0.2 (0.4)	0.1 (0.3)	0.07	0.03	48.2 (11.0)	50.9 (12.9)	−0.23	−0.11
Parents of chronically ill children [15]	147	315	462	6 (4.1)	23 (7.3)	29 (6.3)	0.0 (0.2)	0.1 (0.3)	−0.14	−0.07	45.6 (13.2)	51.3 (14.2)	−0.42	−0.20
**Non-malignant disease**	196	256	452	17 (8.7)	46 (18.0)	63 (13.9)	0.1 (0.3)	0.2 (0.4)	−0.28	−0.14	47.7 (13.5)	54.9 (17.2)	−0.47	−0.23
Overweight persons [16]	12	122	134	4 (33.3)	29 (23.8)	33 (24.6)	0.3 (0.5)	0.2 (0.4)	0.21	0.10	59.9 (16.5)	58.1 (17.8)	0.11	0.05
Paraplegics [17]	147	69	216	10 (6.8)	5 (7.2)	15 (6.9)	0.1 (0.3)	0.1 (0.3)	−0.02	−0.01	47.5 (13.1)	50.5 (14.7)	−0.21	−0.11
Parents of prematurely born children [18]	18	40	58	0	7 (17.5)	7 (12.1)	0.0 (0.0)	0.2 (0.4)	−0.64	−0.31	38.0 (5.9)	49.8 (18.6)	−0.85	−0.39
Survivors of childhood cancer [19]	19	25	44	3 (15.8)	5 (20.0)	8 (18.2)	0.2 (0.4)	0.2 (0.4)	−0.11	−0.05	50.5 (13.1)	59.8 (14.9)	−0.66	−0.31
**Violence**	265	256	521	45 (17.0)	54 (21.1)	99 (19.0)	0.2 (0.4)	0.2 (0.4)	−0.10	−0.05	52.3 (18.1)	57.0 (16.6)	−0.27	−0.13
Assault victims (Aarhus) [20]	138	50	188	42 (30.4)	16 (32.0)	58 (30.9)	0.3 (0.5)	0.3 (0.5)	−0.03	−0.02	57.7 (21.0)	58.5 (16.7)	−0.04	−0.02
Knife homicide at a Danish gymnasium [21]	107	172	279	1 (0.9)	25 (14.5)	26 (9.3)	0.0 (0.0)	0.1 (0.4)	−0.54	−0.26	45.0 (9.4)	55.6 (15.5)	−0.83	−0.38
Robbery victims [22]	20	34	54	2 (10.0)	13 (38.2)	15 (27.8)	0.1 (0.3)	0.4 (0.5)	−0.69	−0.32	54.8 (18.2)	62.4 (20.4)	−0.39	−0.19
**Any trauma**	2101	3119	5220	277 (13.2)	798 (25.6)	1075 (20.6)	0.1 (0.3)	0.3 (0.4)	−0.32	−0.16	27.1 (9.6)	33.0 (9.9)	−0.60	−0.29

Note: N: number of participants; SD: standard deviation; *d*: Cohen’s *d* (effect size); *r:* Pearson’s *r* (effect size); HTQ: Harvard Trauma Questionnaire; PTSD: Posttraumatic Stress Disorder. * In the study column, the parentheses refer to the studies in the reference list. ** In this study only data from the HTQ16 was available. The HTQ16 contains the first 16 items of the HTQ. *** Prospective data gathered through a Danish hospital. **** Data gathered through the National Danish Society of Infant Death.

### Gender difference in the categorical qualification of PTSD

Table 1 provides results about the gender difference in qualification for PTSD. It shows that the gender difference for the entire sample has a solid small effect size (d = 0.32). The gender difference among the trauma types all showed that women qualified for PTSD more easily than men. The highest gender difference in PTSD prevalence was found in *disaster and accident *(*d* = 0.45), showing a prevalence more than two times higher in females than in males, as men showed a prevalence of 14.5% and women a prevalence of 33.2%. For *non-malignant disease* and *loss,* the results showed a small gender difference (*d* = 0.28), whereas, v*iolence* (*d* = 0.10) and *chronic disease* (*d* = 0.08) showed no gender difference in qualification for PTSD.

### Gender differences in the HTQ score

The results for gender differences measured dimensionally by the HTQ total score can be seen in Table 1. The results showed that women had a higher score in HTQ than men with all trauma types. They also showed that the trauma type with the highest gender difference was d*isaster and accident* (*d* = 0.84), which showed a high gender difference. The trauma type with the lowest gender difference was *violence* (*d* = 0.27), which showed a small level of gender difference. *Non-malignant disease* (*d* = 0.47) and *loss* (*d* = 0.47) were close to showing a moderate level of gender difference, while *chronic disease* (*d* = 0.39) showed a smaller gender difference. The effect size for any trauma showed a gender difference of a medium effect (*d* = 0.60).

## Discussion

The present study explores the existence of gender differences in PTSD in relation to different trauma types building on an analysis of a large set of convenience samples. Altogether, this study established that the overall size of the gender difference in PTSD is in line with findings from previous research and shows a two-fold higher risk of PTSD among women than among men. The results established that gender differences exist in the prevalence of PTSD, and that gender differences in PTSD prevalence are not a constant size but varies by trauma type. Gender differences also existed within the examined trauma types although these results were somewhat equivocal. The trauma type in the present study which showed the highest level of gender difference in PTSD was *disaster and accident*, whereas *violence* showed the lowest level of gender difference in PTSD, contradicting previous findings [2]. The same reservation applies to the trauma type *chronic disease* which showed a gender difference when measured by dimensional principles but not when measured by categorical principles. Besides these ambiguities, the size of the gender difference varies across the different trauma types as seen in Table 1.

Also, the present study showed that no linear correlation existed between the prevalence of PTSD and the size of the gender differences in PTSD for the trauma types examined. Thus, a high prevalence of PTSD is not necessarily followed by a high degree of gender difference. This fact calls for considerations regarding special characteristics of the trauma types which for instance determine severity of trauma and PTSD, but it also calls for considerations regarding gender specific characteristics in men and women that influence the vulnerability or resilience to PTSD. This adds to the previously mentioned considerations by Norris et al. suggesting that trauma severity might diminish gender differences in PTSD if the severity exceeds a certain level [26]. Thereby, a possible threshold or ceiling theory is indicated for the effect of trauma severity on gender differences in PTSD. The present study, however, does not support the ceiling theory. A higher level of gender difference exists when estimates are based on dimensional measurement principles than when they are based on categorical measurement.

The present study found that more than every fifth woman and every sixth man experiencing violence qualified for PTSD. Thus, violence is a trauma type connected with a relatively high prevalence of PTSD but at the same time no gender difference was found in the prevalence rates for violence unlike what previous studies have suggested [2,27]. Every fifth to sixth woman experiencing non-malignant disease qualified for PTSD but here only every eleventh to twelfth man showed qualification for PTSD. The prevalence of PTSD for the trauma type non-malignant disease was 13.9% showing an increased risk of PTSD when exposed to non-malignant disease.

### Limitations of the study

The present study is based on data from 18 different convenience samples, and has a total sample of 5220 participants. Thus, all participants had been exposed to a potentially traumatic situation, strengthening that the study examines how women and men react to trauma and how specific trauma types affect men and women. However, certain limitations of the study must be considered. The cross-sectional design of the study raises questions regarding the representativeness of the sample due to the potential unique characteristics of the sample e.g. in geographical and/or socio-economic factors, the risk of possible cohort differences, and the risk of including too specific and unique traumatic events. Thus, the participants in the present study have been selected by convenience, as they were the ones present under a traumatic incident, or in other ways the ones experiencing the trauma. This does not necessarily guarantee that the sample is representative. However, arguments can be made that the size of the total sample strengthens the reliability of the study. Yet, the reliability of the study could have been further improved by including control variables. The lack of control variables must be regarded as a central limitation of the study. The use of self-report questionnaires as the only method of measurement can also be considered as a central shortcoming of the study. Although good reliability and validity has been found for the HTQ [4], the data from self-report questionnaires in general, are met with some reservations.

## Conclusions

The present study establishes that gender differences in PTSD prevalence vary across different trauma types, and women show a higher PTSD prevalence in all trauma types. The gender differences were more pronounced after disaster and accident exposure and smaller after violence and chronic disease. These findings are partly in contrast to previous research. Future research should focus on examining the trauma type related gender difference in PTSD prevalence in order to support the findings from the present study. Future research should also try to verify these findings in other convenience samples as well as in studies based on representative samples. We need more knowledge about causal and relational variables that cause the trauma type related gender differences in PTSD prevalence, to understand why gender differences occur and what influences them in specific contexts. Thereby, interesting findings and important information could be added to the aetiology and phenomenology of PTSD.

## Competing interests

The authors declare that they have no competing interests.

## Authors’ contributions

DD and AE planned the design and did the analyses together. DD drafted the manuscript. Both authors read and approved of the final manuscript.
